# A Biomonitoring Pilot Study in Workers from a Paints Production Plant Exposed to Pigment-Grade Titanium Dioxide (TiO_2_)

**DOI:** 10.3390/toxics10040171

**Published:** 2022-03-31

**Authors:** Enrico Bergamaschi, Valeria Bellisario, Manuela Macrì, Martina Buglisi, Giacomo Garzaro, Giulia Squillacioti, Federica Ghelli, Roberto Bono, Ivana Fenoglio, Francesco Barbero, Chiara Riganti, Antonella Marrocco, Sara Bonetta, Elisabetta Carraro

**Affiliations:** 1 Department of Public Health and Pediatrics, University of Torino, Via Pietro Giuria, 10126 Torino, Italy; enrico.bergamaschi@unito.it (E.B.); valeria.bellisario@unito.it (V.B.); manuela.macri@unito.it (M.M.); martina.buglisi@unito.it (M.B.); giacomo.garzaro@unito.it (G.G.); giulia.squillacioti@unito.it (G.S.); federica.ghelli@unito.it (F.G.); sara.bonetta@unito.it (S.B.); elisabetta.carraro@unito.it (E.C.); 2 Department of Chemistry, University of Torino, Via Pietro Giuria, 10124 Torino, Italy; ivana.fenoglio@unito.it (I.F.); francesco.barbero@unito.it (F.B.); 3 Department of Oncology, University of Torino, 10124 Torino, Italy; chiara.riganti@unito.it; 4 Department of Environmental Health, Harvard T.H. Chan School of Public Health, Boston, MA 02115, USA; amarrocco@hsph.harvard.edu

**Keywords:** paints production, TiO_2_ powders, biological monitoring, exhaled breath condensate, oxidative stress, occupational health

## Abstract

Among particulate matter composing paints, titanium dioxide (TiO_2_) forms about 20% of the final suspension. Although TiO_2_ is broadly used in many applications, TiO_2_ powders represent an established respiratory hazard for workers with long-term exposure. In 35 workers of a paints production plant (15 exposed and 20 not exposed), we assessed pro-inflammatory cytokines (IL-1β, TNF-α, IL-10, IL-17), surfactant protein D (SP-D) and Krebs von den Lungen-6 glycoprotein (KL-6) in exhaled breath condensate (EBC). In urine samples, we measured 8-isoprostane (Isop) and Malondialdehyde (MDA) as biomarkers of oxidative stress, and Titanium (Ti-U) as a biomarker of exposure. Health status, habits and occupational history were recorded. Airborne respirable dusts and Ti were quantified. Particle number concentration and average diameter (nm) were detected by a NanoTracer™ monitoring device. Ti was measurable in filters collected at the respiratory breathing zone (0.11–0.44 µg/m^3^ 8-h TWA). IL-1β and IL-10 values were significantly higher in exposed workers, whereas SP-D was significantly lower (*p* < 0.001). KL-6 was significantly higher in workers than in controls (*p* < 0.01). MDA levels were significantly increased in exposed workers and were positively correlated with Ti-U. Exposure to TiO_2_ in paint production is associated with the subtle alterations of lung pathobiology. These findings suggest the need for an integrated approach relying on both personal exposure and biomarker assessment to improve the hazard characterisation in occupational settings.

## 1. Introduction

Paint manufacturing involves different operations, such as powder handling, pouring, mixing, dispersing, bin filling and cleaning [1]. Huge volumes of the materials added in conventional paints are handled as dry powders, and some studies have revealed that large amounts of ultrafine particles (UFPs) can be generated during the handling of conventional materials [2,3], which may fall under the EU definition of nanomaterials or contain a fraction of nanoparticles. As a result, during material processing, workers are exposed to a heterogeneous mixture of different particles, which can make the quantitative exposure characterisation and risk assessment very complex. Recent studies have shown that the release of UFPs originating from the handling of conventional micron-sized materials may be substantial [4].

Many of the pigments and fillers used in paints can cause diverse negative health effects after lung exposure, e.g., [5,6,7], even though both the technological development and the compliance of companies to safety requirements have contributed to dramatically lowering exposure levels.

Among particulate matter composing paints, titanium dioxide (TiO_2_) may represent more than 20% of the final suspension by weight. TiO_2_ has been used for decades in a wide range of applications, such as photocatalysis, cosmetics and pigments for paints, mainly due to its ability to confer whiteness and opacity to these products. Its high technological attractiveness originates from its light-scattering properties and very high refractive index, which means that relatively low levels of the pigment are required to achieve a white, opaque coating, with the range of light scattered depending on particle size [8].

Although for many years TiO_2_ has been considered as an inert and safe material, belonging to low-solubility and low-toxicity particles (LSLTP), with the increasing production of ultrafine TiO_2_ powders, concern about the possible consequences of workplace exposure has emerged [9].

The European Chemical Agency (ECHA) has recently classified some TiO_2_ powders, and powder mixtures containing TiO_2_, as carcinogenic by inhalation (Category 2, H351) when supplied on its own or in mixtures, where the substance or mixture contains 1% or more TiO_2_ particles with an aerodynamic diameter <10 µm. Liquid mixtures containing TiO_2_ are not classified as carcinogenic, but if they contain particles with an aerodynamic diameter of <10 µm, they need to be labelled as “hazardous respirable droplets may be formed when sprayed” (EUH211) [10].

The hazard of nanoscale or ultrafine TiO_2_ in occupational settings has been investigated in some studies concerning early health effects in workers manufacturing TiO_2_ [11,12,13,14]. Pelclova et al. [13,15] performed a study of 36 workers exposed to NP-TiO_2_ pigment and 45 controls. The median total masses of TiO_2_ concentrations measured in manufacturing laboratories in 2012 and 2013 were 0.65 and 0.40 mg/m^3^, respectively. The median of the concentrations measured by the scanning mobility particle sizer (SMPS) and aerodynamic particle sizer (APS) were 1.98 × 10^4^ and 2.32 × 10^4^ particles/cm^3^, respectively, with about 80% of particles smaller than 100 nm in diameter. Most of the oxidative stress biomarkers, assessed in the exhaled breath condensate (EBC) samples, taken before the start and at the end of the shift, were higher in production workers than in the control groups. A multiple linear regressions analysis confirmed the association between the production of TiO_2_ and the levels of the biomarkers. The Ti concentration was determined in EBC as a direct biomarker of the exposure of workers involved in the production of the TiO_2_ pigment, while the biomarkers of oxidative stress reflected the biological effects of NP-TiO_2_ on the respiratory tract. In a further study, Pelclova et al. [13] focused on lipid peroxidation biomarkers both in EBC and urine to identify the most suitable oxidative stress markers for the non-invasive routine biological monitoring of workers occupationally exposed to NP-TiO_2_. Malondialdehyde (MDA), 4-hydroxy-trans-hexenal (HHE), 4-hydroxynonenal (HNE), 8-isoprostaglandin F2α (8-ISO) and linear aldehydes (C6-C12) were determined in the EBC and urine of 34 workers and 45 unexposed controls. All lipid peroxidation biomarkers were significantly higher in workers than in controls. Moreover, a dose-dependent association was found between TiO_2_ and lipid peroxidation biomarkers in EBC, but not in urine samples. These results confirm the oxidative stress hypothesis as the main mechanism of action of TiO_2_ and give a consistent explanation of the lung damage occurring after long-term exposure.

Tumor necrosis factor alpha (TNF-α) and interleukins 1-beta (IL-1 β) are cytokines activated in the presence of oxidative stress, as they are directly up-regulated by the redox-sensitive transcription factor nuclear factor-κB (NFκB) and/or increased upon the activation of inflammosome [16]. An increase in the level of TNF-α and IL-1 β is indicative of the recruitment and activation of pro-inflammatory Th1 lymphocytes [17]. In addition, IL-17 is produced upon pro-inflammatory stimuli to indicate the recruitment of macrophages and Th2 lymphocytes to promote the maturation of B lymphocytes in plasma cells [18]. By contrast, IL-10 is an immuno-suppressive cytokine, which reduces the recruitment of effector T cells and counteracts the effects of TNF-α and IL-1β. It is common that the exposure to pro-oxidant agents simultaneously increases biomarkers of oxidative stress such as MDA, HHE, HNE and 8-ISO, and inflammatory cytokines [16]. These mechanisms may trigger a vicious circle that amplifies the initial damage on the lung tissue, leading to necro-apoptosis or, in the case of prolonged oxidative stress and chronic inflammation, fibrosis [16].

Zhao et al. [14] carried out a cross-sectional study in a nano-TiO_2_ manufacturing plant in eastern China. Besides TiO_2_ exposure, the authors assessed some biomarkers of cardiopulmonary effects in exposed workers and in control subjects. In the packaging workshop, the total mass concentration of particles (measured by a micro-orifice uniform deposit impactor in a range 10–18,000 nm) was 3.17 mg/m^3^, with 39% of nanoparticles accounting for the whole mass. Lung damage markers (namely, surfactant protein D—SP-D); cardiovascular disease markers (VCAM-1, ICAM-1, LDL, and TC); oxidative stress markers Superoxide Dismutase (SOD) and MDA; and inflammation markers (IL-8, IL-6, IL-1β, TNF-α, and IL-10) showed association with TiO_2_ nanoparticles’ concentration. Similarly, SP-D showed a (dose)-response relationship within the exposed group.

The above studies were carried out in production facilities, where high levels of exposure are expected, whereas no studies are available among TiO_2_ end-users, which usually experience lower exposure levels. Moreover, the subtle changes observed in previous studies need to be further confirmed at lower exposure concentrations.

One existing gap is the lack of information regarding the physico-chemical and morphological characterisations of the TiO_2_ used in the various production processes. In fact, the most recent epidemiological studies suggest the need to adequately characterise TiO_2_ powders to understand which of them can cause harmful effects [19].

With the aim to fill in these gaps, we carried out a pilot study aimed at characterising the exposure to TiO_2_ in an occupational setting and testing a battery of biomarkers of local pulmonary or systemic effects in a small group of occupationally exposed workers during the production of paints.

## 2. Materials and Methods

### 2.1. Subjects and Study Design

The study was carried out in a company producing paints in the province of Turin, north western Italy. A workplace site visit was carried out to assess the exposure to powders, including TiO_2_, and to identify the critical production phases. Therefore, 35 workers were recruited: 15 exposed workers assigned to the production departments and 20 not-exposed workers (control group: administration, design, and marketing). All the workers were informed about the study through an information brochure on the aims, objectives and the various phases of the project aims. Each subject was identified and anonymised with a unique numeric code, and all data, in accordance with current EU legislation, have been processed in an aggregate and anonymous manner. The study was conducted according to the guidelines of the Declaration of Helsinki and approved by the Bioethics Committee of the University of Turin (protocol number 256219/2019). A format to gather the informed consent to participate in the study was signed by all the participants upon the approval by the Bioethical Committee. A questionnaire was administered to all workers involved to collect information about working exposure/habits and on some possible confounding factors (e.g., age, personal and work history, drug use, smoking and alcohol habits, etc.).

### 2.2. Characterisation of Materials Containing TiO_2_

The two TiO_2_ powders that were mainly used as pigment in the factory are herein indicated as (i) T-PS: a pure, untreated TiO_2_; (ii) T-PR: a TiO_2_ with traces of Al and Si and an organic treatment. The commercial names are not reported so as to not identify the supplier. All samples were characterised for elemental composition, size distribution, morphology, crystal structure, surface charge and surface reactivity.

Size distribution in the micrometric range. An analysis was performed by using a Sysmex FPIA3000 analyser. Before the measurements, the samples were suspended in water (0.5 mg/mL) and sonicated for 2 min with a probe sonicator (100 W, 60 kHz, Sonoplus, Bandelin, Berlin, Germany). A high-power field (2× secondary lens) was applied allowing us to measure particles from 1 to 40 μm.

Size distribution in the nanometric range. An analysis was performed by using a ZetaView^®^ PMX-120 (Particle Metrix GmbH, Inning am Ammersee, Germany) nanoparticle tracking analyser (NTA), equipped with a light source wavelength of 488 nm. Before the measurements, the samples were suspended in double filtered milli-Q water (5 mg/mL) and well vortexed, then stock dispersions were further diluted in double filtered milli-Q water (final concentration 5 × 10^−5^ mg/mL); the concentration was found suitable for the NTA analysis. After the optimisation of the instrumental parameters, the sensitivity and the shutter were set at 65 and 100, respectively; 33 videos of 1 s for each sample were recorded analysing ~50 particles/video.

Crystalline phase. X-ray diffraction (XRD) spectra measurements were performed by means of a diffractometer (PW1830, Philips, Amsterdam, The Netherlands) using CoKa radiation, in the (20–90) 2q range, with step width 2q ¼ 0.05. Diffraction peaks have been indexed according to the ICDD database (International Centre for Diffraction Data). The spectra have been elaborated (X’pert Highscore 1.0c, PANalytical B.V.) in order to assess the crystalline phase of the different specimens.

Specific surface area (BET). The surface area of the particles was measured by means of the Brunauer, Emmett, and Teller (BET) method based on N_2_ adsorption at 77 K (Micrometrics ASAP 2020).

Surface chemistry. The ζ-potential was evaluated by means of electrophoretic light scattering (ELS) (Zetasizer Nano-ZS, Malvern Instruments, Worcestershire, UK). TiO_2_ particles were suspended in ultrapure water and then sonicated for 2 min with a probe sonicator (100 W, 60 kHz, Sonoplus, Bandelin, Berlin, Germany). The ζ-potential was measured at different pH (2–9) by adding 0.1 M HCl or NaOH to the suspension.

Surface reactivity. The NMs surface reactivity was monitored by EPR spectroscopy (Miniscope 100 EPR spectrometer, Magnettech, Berlin, Germany) using TEMPONE-H (1-hydroxy-2,2,6,6-tetramethyl-4-oxo-piperidine, Enzo Life Sciences, Inc., New York, NY, USA) as the spin probe. An amount of powder, corresponding to an exposed surface area of 1.4 m^2^, was suspended in 2 mL of water or phosphate buffer containing TEMPONE-H 50 mM (1-hydroxy-2,2,6,6-tetramethyl-4-oxo-piperidine, Enzo Life Sciences, Inc., New York, NY, USA). The suspension was constantly stirred under illumination in a quartz vial equipped with a 500 W mercury/xenon lamp (Oriel Instruments, Stratford, CT, USA) and an IR water filter, to avoid the overheating of the suspensions and a 400 nm cut-off filter. The EPR spectra were recorded on 50 μL of the suspension.

EDX analysis. A qualitative elemental analysis was performed by scanning electron microscopy (SEM—COXEM EM30AX Plus EDAX, Daejeon, Korea).

### 2.3. Environmental and Personal Monitoring

Airborne dust concentrations were measured at specific workstations identified during the site visit. In particular, departments corresponding to specific operations have been identified: (a) water-based paints and storage; (b) enamels and solvents; (c) administrative offices; (d) quality control laboratory; (e) packaging; (f) warehouse. Among these areas, those characterised by greater production activity were chosen for environmental and personal sampling.

The exposure of workers was assessed by environmental and personal sampling, determining the mass, number of fine/ultrafine particles and physicochemical characteristics (shape, size, crystalline form and reactivity) of the powders containing TiO_2_. Air Check Touch fixed cyclone head samplers (SKC, PA) equipped with polycarbonate filters (0.2 µm cut-off) were used to sample the air directly breathed by workers (personal sampling) as well as near the main processing areas (environmental sampling).

The measurements were integrated with air sampling in the major production areas of the company, where TiO_2_ is usually used, using a Nanotracer^TM^ PNT1000 particle counter (Oxility, Bilthoven, The Netherlands). The instrument, based on the electric charge of the airborne particles, allows the collection of information concerning these particles in the 10–300 nm dimensional range (nanoparticles and ultrafine particles). Data stored were analysed by the NanoReporter^TM^ software. On each of the two days of the study, we measured the particle number concentration (#particles/cm^3^), the mean particle diameter (nm) and the surface area of lung deposition, i.e., an estimate of the surface area of the particles deposited in the various compartments of the respiratory tract per unit of inhaled air volume (µm^2^/cm^3^).

### 2.4. Biological Sampling

Biological sampling involved the collection of the exhaled breath condensate (EBC) for the measurement of both oxidative stress and inflammation biomarkers, following the recommendations of the American Thoracic Society and the European Respiratory Society Task Force [19,20]. The EBC was collected for each subject by means of a portable condenser (TURBO-DECCS^TM^—Medivac, Parma, Italy). The TURBO is a refrigeration device and incorporates an aluminium Peltier cooling unit, which houses the EBC collection tube. DECCS refers to the “disposable medical plastic respiratory system”—medical polyethylene—consisting of a mouthpiece connected to a one-way suction valve with a saliva trap and an EBC collection tube inserted into the cooling unit set at −10 °C. Subjects included in the study were asked to breathe at tidal volume into a tube through a mouthpiece with a two-way breathing valve to separate inspiratory and expiratory air and saliva, while wearing a nose clip. On average, 1.7 to 2.3 mL of condensate was collected per participant. Internal standardisation was carried out by collecting a predetermined volume of air (90 L) within about 15 min of breathing at rest by VOLMET 20, suitable for measuring the total volume of air exhaled during an EBC collection session, placed on the DECCS disposable collection circuits. Immediately after the sampling, the EBC samples were divided into 200 μL aliquots in sterile Eppendorf^TM^ tubes and stored at −20 °C for short-term storage (<30 days) and at −80 °C for long-term storage (>30 days).

In parallel, several compounds were analysed as biomarkers of early and subclinical effects in EBC. Tumour necrosis factor alpha (TNF-α), interleukins 1-beta (IL-1β), 17 (IL-17) and 10 (IL-10) were determined as biomarkers of inflammation and immunosuppression. Surfactant protein D (SP-D) and Krebs von den Lungen 6 (KL-6) glycoprotein were determined as potential biomarkers of interstitial lung disease. All analyses were performed with specific immune-enzymatic methods (ELISA).

Finally, before starting the morning work shift, the volunteers provided a spot urine sample for the determination of urinary Titanium (Ti-U), as a biomarker of exposure, and of oxidative stress biomarkers, namely 8-Isoprostane (IsoP) and Malondialdehyde (MDA). Ti-U was determined by inductively coupled plasma mass spectrometry (ICP-MS). Urinary 15-F2t-IsoP concentrations were measured by a competitive enzyme-linked immunoassay (ELISA, Oxford, MI, USA), while MDA concentrations were measured by a colorimetric assay (Oxford, MI, USA), according to the manufacturer’s instructions and as already described in our previous work [21]. The 15-F2t-Isop concentrations were normalized by creatinine. Urinary creatinine was determined by the kinetic Jaffé method.

### 2.5. Statistical Analysis

A statistical analysis was conducted using SPSS for Windows^®^, Version 25.0 (Statistical Package for Social Sciences, Chicago, IL, USA). The normality of the distribution was assessed by the Kolmogorov–Smirnov test. The differences between the central tendency values of the two subgroups examined were evaluated by the Mann–Whitney test and Student’s *t* test. Values are here reported as mean ± standard deviation (SD) and as median and interquartile range (25–75th percentile) for the parametric and non-parametric variables, respectively. The correlations between the variables were evaluated using the Pearson correlation coefficient (r) and Spearman’s rho for the normally distributed and non-normally distributed variables, respectively. Values of *p* < 0.05 were considered statistically significant. A multiple linear regression analysis model, adjusted by smoke and working age, was applied to assess the association between the biomarkers and workplace exposure.

## 3. Results

### 3.1. Characterisation of the Exposure

**Figure 1:**
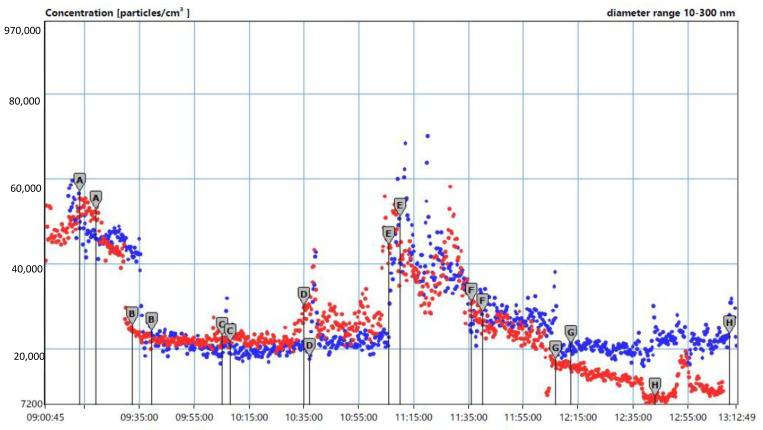
Time course (*X*-axis) of particle number concentrations (*Y*-axis) in the 10–300 nm dimensional range: first day (blue bullet) and second day (red bullet) of the sampling. A–H markers refer to the different working areas assessed in two subsequent days. A = TiO_2_ storage area (confined area used as positive control); B = water-based paint system; C = water-based paint system during automatic bin filling; D = water-based paint system during manual bin filling; E = manual handling of powders, mixing and dispersion; F = manual handling, mixing and dispersion; G = office building; H = outdoor environment (NB: the first day there was one truck to supply the silos with TiO_2_).

Environmental sampling: the time course of particle number concentrations in the 10–300 nm dimensional range was measured by a NanotracerTM PNT1000 particle counter to estimate the emission profile over two standard working days (Figure 1).

The median value of the respirable fraction of dusts was 0.064 mg/m^3^ in the water-based paint production area, while the highest values were found in the enamel production area (0.112–0.137 mg/m^3^). The values of the respirable fraction of airborne TiO_2_ were lower, with peaks of 0.114 µg/m^3^ as the weighted average concentration over an 8 h working time (8 h-TWA) in stationary samples. On the contrary, the TiO_2_ concentrations (8 h-TWA) measured in the workers’ breathing zone showed slightly higher values, with a minimum of 0.011 µg/m^3^ up to values of 0.462 µg/m^3^ detected during the production of water-based paints.

The number of particles in the nanometer range was on average five times greater than the environmental background measured outside the company, during the operations of dumping bags into containers and mixing wet powders. The time course of particle number concentration was quite similar in the two days, owing to quite standardised activities and working tasks.

Table 1 summarises the concentration values in mass units and numbers measured in the various areas during the production process.

### 3.2. Physico-Chemical Characterisation of the TiO_2_ Powders Handled by the Workers

Physico-chemical properties are known to modulate the hazard of particulate materials. Crystallinity [22], surface reactivity [23] and particles’ size and shape [24] have been reported to modulate the toxicity of TiO_2_ powders. To predict the possible properties of the airborne TiO_2_, two TiO_2_ powders used in the factory were analysed. Samples were characterised for composition (EDX), size and dimensional distribution in both nano- and micron-range (NTA, FPIA), morphology (FPIA), crystal structure (X-ray diffractometry), specific surface area (BET), and surface charge (ELS) and surface reactivity (EPR). Table 2 summarises the results.

**Figure 2:**
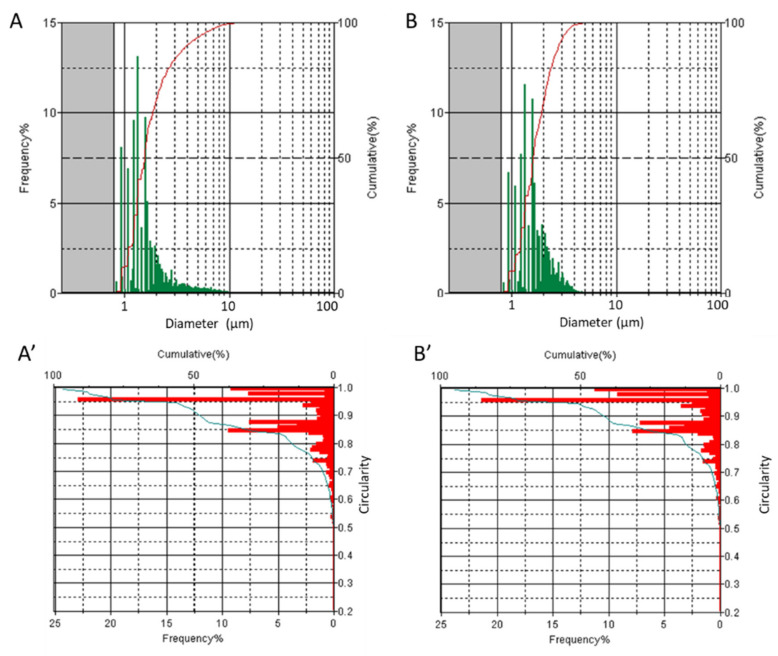
Particle size and shape distribution evaluated by flow particles images analyser (FPIA). Particle size distribution (frequency and cumulative) of (**A**) Ti-PS and (**B**) Ti-PR; circularity (frequency and cumulative) of (**A’**) Ti-PS and (**B’**) Ti-PR.

Both powders were very electrostatic and able to disperse extremely quickly in the air at minimal contact. This characteristic undoubtedly increases the possibility that they will be absorbed through the respiratory tract by the workers who handle them. The size distribution of the powders was analysed in the micrometric and nanometric range by integrating FPIA (Figure 2) and NTA (Figure S1) techniques.

As expected, both powders contained particles in the micrometric range (Figure 2). However, Ti-PR appeared to have particles in the respirable range (1–4 μm). In total, 50% of the particles had a diameter lower than 2 μm. Ti-PS exhibited particles with a diameter in the whole detectable range (1–10 μm), and, similarly to Ti-PR, 50% of the particles had a diameter lower than 2 μm. Particles appeared to be mainly isometric (50% of the particles had a circularity >0.9), but with an irregular shape, as expected by the powder obtained by grinding. Nanometric particles were detected for both samples (Figure S1) in similar abundance. The nanoparticles covered a wide range of size distribution, and had a mean hydrodynamic diameter of 187.1 and 169.9 nm for Ti-PS and Ti-PR, respectively. The measurement of the specific surface area (SSA) gave complementary information on the particles’ size for non-porous materials. Ti-PS exhibited an SSA of 56.2 + 0.27 m^2^/g typical of a nanometric powder, suggesting that micrometric particles were formed by aggregates of smaller particles. Conversely, Ti-PR had an SSA equal to 14.66 + 0.11 m^2^/g, typical of a micrometric powder.

An XRD analysis revealed rutile as the single crystalline phase for both samples (Figure S2). The properties of the surface of the particles were investigated by electrophoretic light scattering (ELS) measuring the ζ-potential values at different pH (Figure S3A). The ζ-potential curves had the typical features of TiO_2_ powder, with positive ζ-potential values at acidic pH and negative pH at basic pH. However, the two samples had a different point of zero charge (PZC), suggesting the presence at the surface of coating/contaminants. These data suggest that TiO_2_ was exposed at the surface in both samples. This has been confirmed by EPR spectroscopy (Figure S3B). The powders have been put in contact with a probe [25] and activated with a UV lamp. In this condition, the probe reacts with the surface of TiO_2_ generating a radical species that is detected by the EPR spectrometer. Both materials exhibited an intense EPR signal, due to the activation of the TiO_2_ phase in both cases exposed to the surface. The signals were lower than those observed for an anatase/rutile photo-reactive material (Aeroxide P25). This was expected because of the crystalline phase (rutile), and less reactive with respect to anatase. T-PR appeared more reactive than T-PS, despite the lower specific surface area and the presence of an organic treatment. This might be due to the presence of surface contaminants possibly modifying the TiO_2_ reactivity.

### 3.3. General Descriptive of the Population under Study

Table 3 summarises the main general characteristics of the enrolled groups. The two groups were homogeneous for age and for some characteristics (e.g., BMI) (Levene’s test of homogeneity of variance = N.S.). Smokers reported 2–10 cigarettes/day.

Table 4 reports the main general values of oxidative and inflammatory biomarkers, also distinguished by TiO_2_ exposure. The table also reports the results of statistics (Mann–Whitney U test).

**Figure 3:**
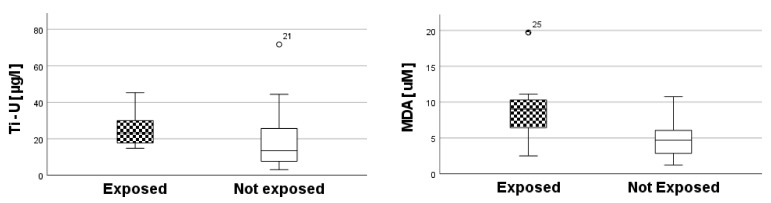
Differences in exposed vs. not exposed of Ti and MDA, as biomarkers of exposure and oxidative stress, respectively.

Among the urinary biomarkers of exposure and oxidative stress, Ti and MDA concentrations were significantly higher in exposed workers than in control subjects (Mann–Whitney: *p* = 0.02 and *p* = 0.002, respectively) (Figure 3), while IsoP was not significantly different between the two groups.

**Figure 4:**
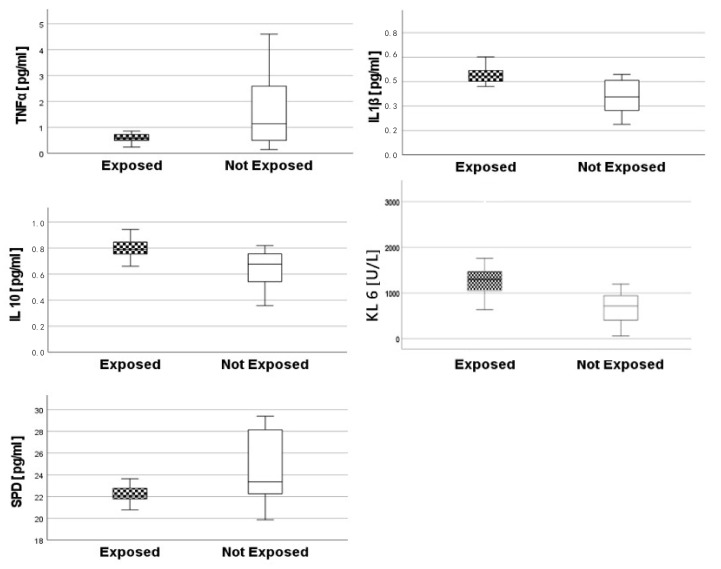
Descriptive statistics of biomarkers determined in EBC (Mann–Whitney test). Pro-inflammatory cytokines TNF-α, IL-1 β and of IL-10 were significantly different between groups (*p* = 0.003, *p* < 0.001, *p* < 0.001, respectively). KL-6 glycoprotein values were significantly higher in exposed workers than in not exposed (*p* = 0.003), whereas SP-D values were significantly lower in exposed workers than in not exposed (p = 0.011).

Figure 4 shows the results of EBC biomarkers in exposed and control workers. Pro-inflammatory cytokines TNF-α, IL-1 β and IL-10 were significantly different between groups (Mann–Whitney: *p* = 0.003, *p* < 0.001, *p* < 0.001, respectively). Conversely, IL-17 showed no significant difference between groups. SP-D values were significantly lower in exposed workers than in controls (Mann–Whitney test: *p* =0.011). Conversely, the KL-6 glycoprotein values were significantly higher in exposed workers than in controls (Mann–Whitney test: *p* = 0.003).

Positive correlations were found between the urinary biomarkers of effects and oxidative stress. In particular, Ti-U concentrations were positively correlated to MDA levels (Spearman’s rho = 0.841, *p* < 0.001). EBC biomarkers also were inter-correlated. In particular, TNF-α was negatively correlated with IL-1β (Spearman’s rho = −0.428, *p* < 0.001), with IL-10 (Spearman’s rho = −0.454, *p* = 0.006) and with KL-6 (Spearman’s rho = −0.391, *p* = 0.02). IL-1β and IL-10 showed a positive and statistically high correlation (Spearman’s rho = 0.705, *p* < 0.001), whereas IL-10 was correlated with KL-6 (Spearman’s rho = −0.477, *p* = 0.004). In addition, IL-1β and KLF6 showed a positive correlation (Spearman’s rho = 0.433, *p* = 0.009). Finally, significant correlations were found also between MDA and TNF-α (Spearman’s rho = −0.402, *p* = 0.02), MDA and IL-1β (Spearman’s rho = 0.449, *p* = 0.007) and MDA and IL-10 (Spearman’s rho = 0.434, *p* = 0.009). Despite the exclusion of different not-significant biomarkers, MDA and IL-1β were chosen as the gold standard of the molecular mechanisms in urine or in the EBC matrix, respectively.

**Figure 5:**
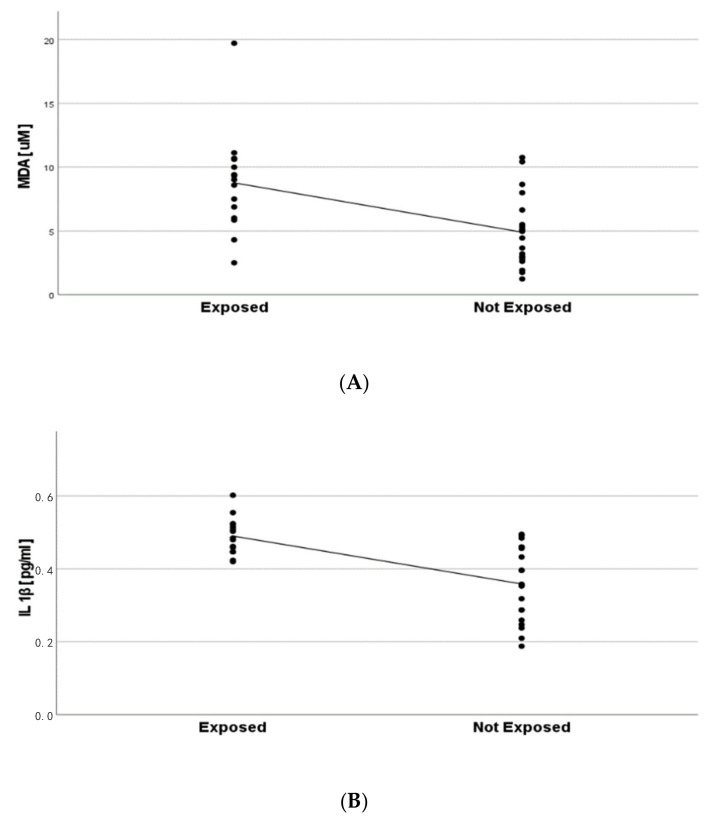
Regression model between working exposure and MDA (urine gold standard) (**A**) and IL1β (EBC gold standard) (**B**).

Regarding the urine matrix, the linear regression model, adjusted by smoke and working age, proved an inverse relationship between MDA levels and the workplace exposure (B = −2.5, *p* = 0.04, CI: −0.065/−0.088) (Figure 5A). In EBC, IL-1β showed a similar significant relationship (B = −0.46, *p* = 0.04, CI: −0.007–0.005) (Figure 5B).

## 4. Discussion

Despite the large quantities of material (tons of pigment) handled daily for the production of paints, the present study revealed considerable compliance with safety procedures in the company under investigation. Indeed, the concentration of TiO_2_ in the production areas was therefore very low, due to the presence of highly efficient capture systems located near the sources of potential emission, and the natural ventilation assured by the huge doors of the factory that were maintained open. As a result, the airborne concentrations of respirable dusts were always much lower than the current occupational exposure limit values proposed by various international agencies. Nevertheless, the concentration of TiO_2_ in the workers’ breathing zone was quantifiable to a modest extent and showed values higher than the airborne concentrations, probably because of the close proximity of workers to the emission sources. The dimensional characterisation of the TiO_2_ powders handled by the workers confirmed the presence of particles in the micrometric range, but also the presence of an ultrafine fraction of particles, as inferred by NTA and, for Ti-PS, BET analyses.

Currently, in Europe, there are no harmonised limits regarding exposure to TiO_2_ dusts neither for workers nor for the general population; however, the growing industrial application of NP-TiO_2_ has led several countries to recommend threshold limit values valid for exposures of 8 h a day, for the entire duration of working life. The National Institute for Occupational Safety and Health (NIOSH) in 2011 recommended different occupational exposure limits (REL = recommended exposure level) for fine TiO_2_ and for ultrafine TiO_2_. The recommended airborne exposure limit (REL) is set at 2.4 mg/m^3^ for fine TiO_2_ and 0.3 mg/m^3^ for ultrafine (including engineered nanoscale) TiO_2_, as the time-weighted average (TWA) [9].

Some research programs have suggested occupational exposure limits, such as ENRHES (Engineered Nanoparticles: Review of Health and Environmental Safety), which defined a DNEL (derived no effect level), i.e., an exposure level above which humans should not be exposed equal to 0.017 mg/m^3^ (17µg/m^3^) for 8 working hours. This value was extrapolated from an NOAEC (no observed adverse effect concentration) corresponding to 5 mg/m^3^ for repeated toxicity in a 13-week inhalation study (6 h a day, 5 days a week, exposed to particles from 21 nm) [26].

The French Agency for Food and the Environmental and Occupational Health and Safety (ANSES) has recommended an 8 h occupational exposure limit of 0.80 μg/m^3^ [27]. Interestingly, the same agency has proposed a toxicological reference value of 0.12 µg/m^3^ for nanosized TiO_2_ used when conducting health risk assessments as part of the management of industrial facilities and sites in France [28]. The above value would represent a benchmark value for the prevention of fibro-proliferative alterations and the progressive alteration of the alveolar epithelium towards bronchiolysis [29,30].

However, this threshold can be applicable only to TiO_2_-P25 powders (consisting of 80% anatase and 20% rutile with a diameter = 21 nm), which is the only TiO_2_ in powder form tested in the study used to establish this value [28]. Considering the huge variety of TiO_2_ powders on the European market (more than 350 different types) and the absence of exhaustive toxicological data for each of these, this value may not be applicable for the other forms of NP-TiO_2_ (due to the different diameter, crystalline structure, surface coating, etc.) [19]. In the present study, the powders were in the rutile form, which is considered less reactive and hazardous compared to anatase [25]. Though one of the samples was declared by the provider to be treated with an organic coating, in both cases, the ELS and EPR measurements revealed TiO_2_ exposed at the surface of the particles.

Over the two days of measurements, some airborne mass concentrations gave values very close to the limit value recommended by the ANSES (0.12 µg/m^3^). Thus, in spite of the very low amount measured, we cannot rule out the possible effect occurring in the long time period. This is the main reason why we have assessed both exposure and biomarkers of effect, measured in accessible biological matrices.

Biological monitoring, a widely used practice in occupational and environmental toxicology, combines the use of exposure indicators that provide information on the level of exposure of the subjects, and biological indicators of the effect that aim to highlight potential early and reversible biological alterations associated with/caused by a given exposure.

Regarding biomarkers of exposure, we were able to quantify the urinary concentration of Titanium (Ti, element, not oxide) as a potential biomarker of exposure and internal dose. Interestingly, we found a substantial stability of the urinary Ti levels measured in spot urine samples of the subjects occupationally exposed at the beginning and at the end of the week (data not reported); the difference between the values at the end and the beginning of the week were negligible.

Toxicokinetic studies of ultrafine/nanoparticles suggest preferential absorption via the respiratory and oral routes with possible systemic translocation that can lead to accumulation in peripheral tissues or excretion via faeces and/or urine [31]. Our findings seem to be in agreement with information about the toxicokinetics of Ti evaluated in experimental animals following single exposure [32,33], suggesting that the kinetics of Ti (especially at low doses) are characterised by a persistence beyond 48 h in the lungs, with a gradual reduction of the pulmonary dose in about two weeks, with a slow urinary excretion. These findings suggest that even lower exposure levels (e.g., by occupation) can account for the difference we found between the exposed and the controls, thus mirroring a sort of “steady state” following the accumulation of Ti, which can also be taken up with the diet. However, a clear-cut meaning in toxicological terms to the amount of Ti measured is not predictable.

In the EBC samples collected from the subjects participating in the study, some biomarkers of effect reflecting morphological or functional alteration in the lung parenchyma have been measured. EBC consists mainly of water vapour condensed from the respiratory tract with small droplets of liquid lining the airways from the respiratory tract, including the bronchial and alveolar regions of the lungs, and it is supposed to mirror lung bio-pathology [20]. Within these droplets, there is an unknown fraction of volatile and non-volatile substances. Volatile substances occur in the form of water-soluble compounds in the condensed water of the EBC sample such as ammonia, hydrogen peroxide and ethanol, while non-volatile substances can include cytokines, lipids and salts, but also environmental contaminants, particles and viruses [20,34].

A common toxicity characteristic of the inhaled particles is their propensity to generate oxidative stress at the cellular level, in relation to their surface reactivity which inversely increases with particle size, and which is therefore exacerbated for nanoparticles. Among the many approaches to assess systemic oxidative stress, the determination of 8-isoprostane (IsoP), is of particular interest because it is a stable molecule, present in many tissues and biological fluids, reflecting lipid peroxidation [35,36,37]. Isoprostanes are prostaglandins generated by cyclooxygenase and by the peroxidation of arachidonic acid. They reliably reflect lipid peroxidation and are ubiquitous in the body, stable in biological fluids and not sensitive to dietary lipid intake. An increase in urinary Isop has been reported after exposure to fine and ultrafine wood smoke particles [37], and in a recent study that aimed to evaluate oxidative stress related to different degrees of urban pollution [17,38].

MDA is another biomarker of lipid peroxidation, which can be easily quantified after the controlled reaction with the reactive substance of thiobarbituric acid (TBARS). Modifications of TBARS concentrations are used to evaluate a wide range of diseases and MDA remains, to date, the most used assay to determine lipid peroxidation [39,40].

IsoP and MDA have been used as biomarkers reflecting systemic oxidative stress, i.e., the oxidative load supported by the organism, and have been found to increase in acute or chronic inflammation and indicate the recruitment and activation of neutrophils and macrophages [41]. However, in our study, urinary IsoP levels were not significantly different between the exposed workers and the control subjects. In spite of this result, we observed that urinary IsoP levels were generally higher if compared with the previous literature [42,43]. This is not surprising, as ELISA assays tend to overestimate IsoP concentrations, mainly due to interferences, and previous authors used liquid chromatography (LC) techniques. Our results are 10-fold higher than those reported by LC-quantified IsoP, falling into the expected range of ELISA overestimation in urine (0.4–61.9 folds) compared to LC [44]. We observed an increase in MDA among the exposed compared to controls, and a positive correlation between the exposure biomarker (Ti-U) and MDA in both the overall group and in the group of exposed workers. This confirms previous observations about an increase in urinary biomarkers of oxidative stress in workers occupationally exposed in the production of pigments [13,45]; although, we found generally lower levels of MDA compared to other studies [43].

In the EBC samples collected from the subjects participating in the study, we measured some biomarkers of effect to assess early functional alteration in the lung biopathology, such as TNF-α, IL-1 β, IL-17 and IL-10 as biomarkers of inflammation and immu-no-suppression. While the increase in IL-1β we detected can be interpreted in a pro-inflammatory sense [17], the significant decrease in TNF-α concentration in the exposed subjects is not easy to interpret. An imbalance between pro-inflammatory TNF-α and anti-inflammatory IL-10 in plasma has been considered a biomarker of suppression of Th1 cell-mediated immunity and exacerbation of Th2 humoral immune response, potentially leading to the development of allergic and autoimmune diseases [46]. We also know that TiO_2_ can increase the cytokine secretion by macrophages in vitro [47]. Whether a similar pattern can occur in lung lining fluids, as reflected by EBC determination, should be further elucidated. Overall, an immuno-suppressive effect seems to prevail, probably linked to a reduced macrophage activity in the airways of the exposed subjects.

Surfactant protein D (SP-D) values were significantly lower in exposed workers than in controls. Although no studies have considered the SP-D in EBC, it is reasonable that these pneumoproteins may be detectable and measurable. SP-A and SP-D belong to a subgroup of type C lectin (molecular weight: 26–36 and 43 kDa, respectively), and are involved in the regulation of pulmonary host defense and inflammation [48,49]. Our findings are consistent with those of Zhao et al. [14], who found a slight but significant decrease in SP-D levels in serum samples of workers occupationally exposed to nano-TiO_2_. These changes were interpreted as the results of a cell injury and/or decrease in number of type II alveolar epithelial cells caused by nanomaterial-induced oxidative stress [14]. In general, it is known that the involvement of the distal airways caused by different types of ultrafine particulates and gases (such as ozone) can induce a reduction in the pneumocyte population, which can explain a reduction in lung and circulating levels of lung pneumoproteins [50].

In our study, conversely, EBC values of the Krebs von den Lungen-6 (KL-6) glycoprotein were significantly higher in exposed workers than in controls. Glycoprotein KL-6 is a high molecular weight mucin (1200 kDa) which has been classified as MUC1—human mucin [51]. Recent clinical studies have suggested that surfactant protein D (SP-D) and Krebs von den Lungen-6 (KL-6) glycoprotein are potential biomarkers of interstitial lung disease. For instance, in Japan they both are used in the clinical setting during the diagnostic tree for the identification of idiopathic pulmonary fibrosis and other types of interstitial lung diseases [52]. The negative correlation between KL-6 and SP-D found in particular in the exposed group seems to suggest a relationship between the depletion of the population of specialized epithelial cells and the activation of a pro-fibrotic cascade. Moreover, these findings are consistent with insights gathered from the mineralogical analyses of BAL samples of patients with lung fibrosis, suggesting a role of titanium nanoparticles in idiopathic pulmonary fibrosis [53].

The overall picture resulting from the combination of the different biomarkers reflecting subtle changes in lung physiology assessed in this study suggests a mild inflammatory status and the activation of a pro-fibrotic cascade. Most likely, these alterations are not due to recent exposure, but could represent a condition due to previous exposure, due to the cumulative effects of such low doses or to the higher exposure occurring in the past. The results also reveal an association between biomarkers of systemic oxidative stress and the exposure of concern. Although not specific, these biomarkers appear to be related to the specific type of exposure investigated.

This biomarker-based approach to assess early effects in workers exposed to nano-objects has been recommended as a component of a strategy to risk assessment and management in the workplace [54,55,56]. In order to enable feasible and acceptable routine screenings in populations at risk, biomarkers that could be analysed in biological matrices collected by non-invasive procedures are of particular relevance. From this perspective, exhaled breath condensates (EBC), exhaled air (EA) and urine are three preferred biological matrices for the non-invasive investigation of biomarkers in workers exposed to UFPs and nanomaterials, as they give insights on local (pulmonary) and systemic oxidative stress and inflammatory response [13,20,21,54,55,56,57,58,59]. Although we recognise the higher performance of the LC-MS/MS technique for the quantification of some biomarkers of oxidative stress as compared to ELISA assays [60,61], we acknowledge that ELISA are less time-consuming and more cost-effective techniques, which do not require particular laboratory equipment and highly-trained operators. This may be considered both a limitation and a strength of this study aimed at supporting an extensive quantification of oxidative stress and inflammatory biomarkers in non-invasively collected specimens to monitor workers’ health in contexts other than research settings, e.g., periodical health surveillance.

## 5. Conclusions

Exposure to TiO_2_ containing dusts well below the occupational exposure limits (OELs), but close to the threshold for preventing fibro-proliferative and progressive alteration of epithelium, can result in subtle lung changes, as reflected by changes in KL-6 and SP-D and an increase in biomarkers of oxidative stress. Interestingly, the lower threshold proposed by ANSES for TiO_2_ represents the benchmark dose for preventing fibro-proliferative and progression alteration of epithelium and alveolar bronchiolisation.

With regard to the exposure parameter, the setting investigated is probably representative of a series of small companies that adopt similar production processes, suggesting a widespread micro-pollution mainly linked to operational but temporary tasks. At the concentrations found in such an environment, however, no short- or long-term effects are expected, owing to the compliance with the threshold limit values adopted or proposed for workplaces. We could argue that just quantifiable aerosols in the nanometer range could affect some health endpoints for which the current standards are not (yet) applicable.

This study suggests the need for a combined approach relying on both exposure assessment and biomarkers of effect to improve the risk assessment in occupational settings in which TiO_2_ is handled, though under strict and effective control measures.

Thus, in spite of several drawbacks in implementing an integrated strategy [54], biomonitoring enables regular exposure and health assessment and can support effective risk management [54,55,56]. Owing to the small number of subjects evaluated and the intrinsic variability of biomarkers, the observed changes along with their health significance must be assessed in a long-term perspective.

## Figures and Tables

**Table 1:** Results of the sampling of airborne dust in the different production environments. PBZ: personal breathing one.

Company Area/ Type of Samplig	Water-Based Paint System	Automatic Bin Filling	Mixing and Dispersion	Administrative Office	Outdoor (Day 1)
Area monitoring respirable dusts (mg/m^3^ 8 h-TWA)	0.064	0.013	0.112; 0.137	0.033	
Area monitoring respirable Ti (µg/m^3^ 8 h-TWA)	0.018	0.018; 0.114	0.012; 0.024	0.013	
PBZ-Ti (µg/m^3^ 8 h-TWA)	0.104; 0.462	0.011; 0.012	0.07; 0.014	0.012	
Particle number concentrations × 10^3^ (average aerodynamic diameter, nm)	24.98; 54.68 (64–73)	20.8; 27.68 (78–93)	40.72; 46.40 (67–95)	16.97 (71)	8.16 (72)

**Table 2:** Elemental composition, crystallinity, average size (hydrodynamic diameter), dimensional distribution and polydispersion index (PdI) of the various samples of TiO_2_.

TiO_2_ Sample	Elemental Composition (% *w*/*w*)	Crystal Phase	Particles Size (FPIA) (μm)	Size (Hydrodynamic Diameter, NTA) (nm)	Surface Area BET m^2^/g	ζ Potential (Water) (mV)
T-PS (untreated TiO_2_)	Ti 58.7, O 40.2, Al 1.1	**100% rutile**	1–4	187.1 ± 118.6	56.2 ± 0.27	−22.7 ± 0.642
T-PR (Al, Si, organic treatment)	Ti 55.0, O 40.9, Al 2.3, Si 1.8	**100% rutile**	1–10	169.9 ± 96.6	14.66 ± 0.11	11.9 ± 1.13

**Table 3:** General descriptive of the population under study. No differences between groups were detectable (Levene’s test of homogeneity of variance). Values are mean ± SD.

Main General Characteristics of the Population Investigated							
	General Sample		Exposed (No. 15)		Controls (No. 20)		Levene’s Test
**Height (cm) ** **Mean ± SD**	174 ± 8.1		175 ± 8.9		173 ± 8		0.4
**Weight (Kg) ** **Mean ± SD**	80.7 ± 14.5		85.7 ± 14.5		82.3 ± 13.6		0.8
**BMI ** **Mean ± SD**	26.6 ± 4.1		27.9 ± 3.5		26 ± 4.4		0.5
**Age (years) ** **Mean ± SD**	47.3 ± 11.5		48.7 ± 10.05		45.9 ± 12.18		0.2
**Working exposure (years) ** **Mean ± SD**	13.8 ± 10.9		14.3 ± 10.8		13.5 ± 12		0.4
**Smoke habits ** **(N.)**	YES	NO	YES	NO	YES	NO	0.1
	9	26	6	9	3	17	

**Table 4:** General descriptive biomarkers measured in EBC and urine sample with Mann–Whitney U test results.

EBC				
	Total	Exposed	Not Exposed	Mann-Whitney U Test
**TNF-α [pg/mL] ** **Mean ± SD, median, range, IQ**	1.6 ± 1.1 0.7; [0.5–1.7]; 1.2	0.6 ± 0.2 0.6; [0.5–0.7]; 0.3	1.6 ± 1.3 1.1; [0.6–2.6]; 2	**0.03**
**IL-1β [pg/mL] ** **Mean ± SD, median, range, IQ**	0.4 ± 0.1 0.45; [0.3–0.5]; 0.1	0.5 ± 0.05 0.48; [0.4–0.5]; 0.07	0.3 ± 0.1 0.35; [0.25–0.45]; 0.2	**<0.001**
**IL-10 [pg/mL] ** **Mean ± SD, median, range, IQ**	1.6 ± 1.1 0.7; [0.5–1.7]; 1.2	0.8 ± 0.07 0.8; [0.7–0.8]; 0.09	0.6 ± 0.1 0.65; [0.5–0.7]; 0.2	**<0.001**
**IL 17 [pg/mL] ** **Mean ± SD, median, range, IQ**	1.1 ± 1.1 1.12; [1–1.2]; 1.12	1.1 ± 0.07 1.1; [1–1.1]; 0.09	1.1 ± 1.4 1.15; [1.1–1.2];0.12	**0.11**
**KL-6 [U/L] ** **Mean ± SD, median, range, IQ**	990 ± 670 820; [620–1210]; 590	1370 ± 780 1190; [780–1470]; 690	690 ± 390 750; [300–1050]; 750	**0.003**
**SPD [pg/mL] ** **Mean ± SD, median, range, IQ**	23.7 ± 2.7 22.8; [21.9–24.4]; 2.4	22.2 ± 0.8 22; [21.7–22.9]; 1.2	24.9 ± 3.2 23.4; [22.2–28.6]; 6.4	**0.011**
**URINE**				
**MDA [µM] ** **Mean ± SD, median, range, IQ**	6.6 ± 3.8 5.7; [3.1–9.3]; 6.2	8.8 ± 3.9 9; [6–10.6]; 4.6	4.8 ± 2.8 4.4; [2.8–5.5]; 2.7	**0.002**
**ISOP [ng/mg crea]** **Mean ± SD, median, range, IQ**	3.7 ± 1.2 3.8; [2.7–4.4]; 1.8	3.9 ± 1.5 3.9; [2.6–4.6]; 2	3.6 ± 1 3.5; [2.7–4.2]; 1.6	**0.5**
**Ti-U ** **Mean ± SD, median, range, IQ**	20.4 ± 16.2 17.9; [1.72–90.4]; 17.2	25.9 ± 19.3 23.3; [10.6–90.4]; 15.7	14.5 ± 10.5 11.9; [1.72–44.4]; 17.9	**0.02**

## Data Availability

Not applicable.

## Supplementary Materials

The following supporting information can be downloaded at: https://www.mdpi.com/article/10.3390/toxics10040171/s1. Figure S1: Particles size distribution evaluated by Nanoparticle Tracking Analysis (NTA). (A) Aqueous dispersion of T-PS. (B) Aqueous dispersion of T-PR. Figure S2. Crystalline phase. XRD patterns of (a) T-PS and (b) T-PR. Figure S3. ζ-potential and surface reactivity. (A) ζ-potential values measured at different pH of T-PS and T-PR; (B) Surface reactivity of T-PS and T-PR evaluated by EPR/spin trapping technique. Ctrl−. no powder; ctrl+ Aeroxide P25.
