# Supplementary material for: A Biomonitoring Pilot Study in Workers from a Paints Production Plant Exposed to Pigment-Grade Titanium Dioxide (TiO2)
Source: Toxics. 2022 Mar 31;10(4):171. doi: 10.3390/toxics10040171 (PMC9028136; doi:10.3390/toxics10040171)
Supplement: Supplementary file 1 [file toxics-10-00171-s001.zip › toxics-1626394-supplementary.pdf]

toxics

Supplementary Materials: A Biomonitoring Pilot Study in Workers from a Paints Production Plant Exposed to Pigment-Grade Titanium Dioxide (TiO_2)

Enrico Bergamaschi, Valeria Bellisario, Manuela Macrì, Martina Buglisi, Giacomo Garzaro, Giulia Squillacioti, Federica Ghelli, Roberto Bono, Ivana Fenoglio, Francesco Barbero, Chiara Riganti, Antonella Marrocco, Sara Bonetta and Elisabetta Carraro

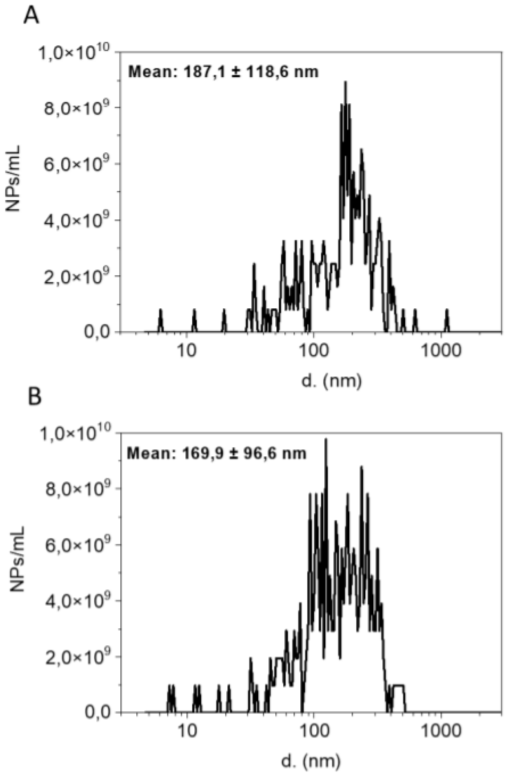

Figure S1. Particles size distribution evaluated by Nanoparticle Tracking Analysis (NTA). (**A**) Aqueous dispersion of T-PS. (**B**) Aqueous dispersion of T-PR.

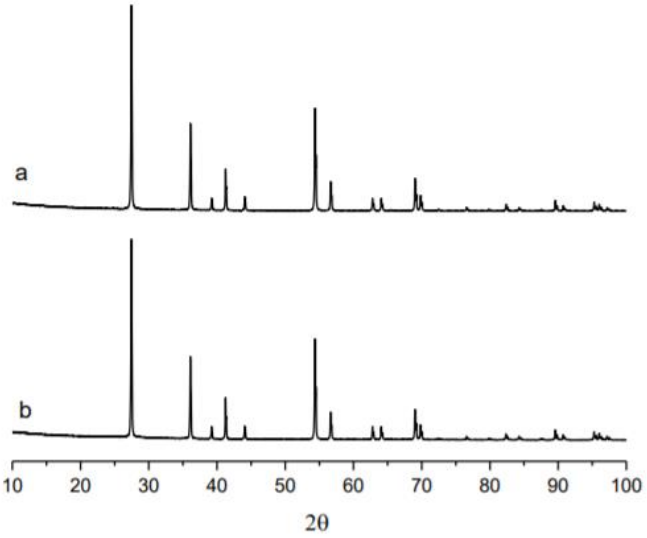

Figure S2. Crystalline phase. XRD patterns of (**a**) T-PS and (**b**) T-PR.

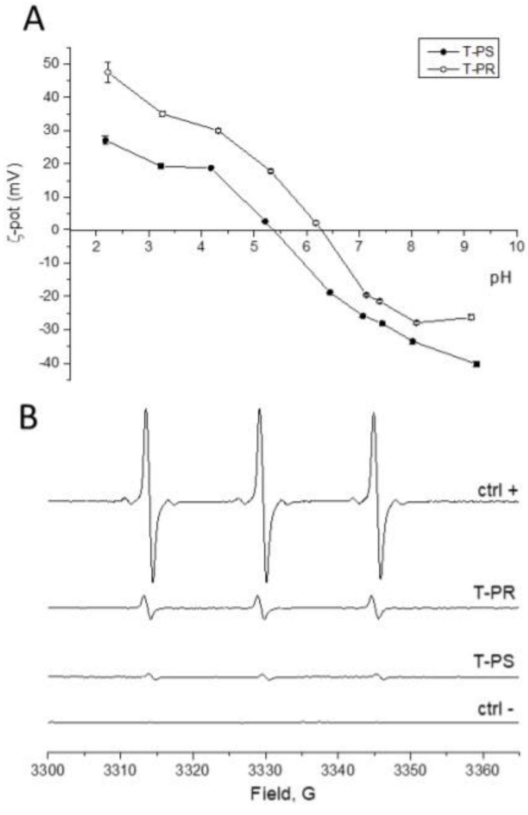

Figure S3. ζ-potential and surface reactivity. (**A**) ζ-potential values measured at different pH of TPS and T-PR; (**B**) Surface reactivity of T-PS and T-PR evaluated by EPR/spin trapping technique. Ctrl - . no powder; ctrl+ Aeroxide P25.
